# Antiresorptive agents enhance ossification of free flap reconstructions of the mandible: a radiological retrospective cohort study

**DOI:** 10.3389/fonc.2024.1401165

**Published:** 2024-06-12

**Authors:** Maximilian Gottsauner, Johannes Meier, Jonas Eichberger, Stephanie Eckmüller, Johannes Schuderer, Mathias Fiedler, Michael Maurer, Torsten E. Reichert, Tobias Ettl

**Affiliations:** Department of Oral and Maxillofacial Surgery, University Hospital Regensburg, Regensburg, Germany

**Keywords:** ossification, MRONJ, antiresorptive agent, microvascular reconstruction, jaw, mandible, fibula

## Abstract

**Background:**

The aim of this study was to investigate the effect of antiresorptive agents on the ossification of reconstructed mandibles by free bone grafts for the first time.

**Methods:**

A total of 38 reconstructions of the jaw were retrospectively evaluated for ossification between bone segments by two raters based on postoperative panoramic radiographs. The study group (n = 13) had segmental resection of the mandible and free bone flap reconstruction due to medication-related osteonecrosis of the jaw (MRONJ). The control group (noMRONJ, n = 25) comprised segmental mandibular resections and free bone flap reconstructions due to tumors, chronic osteomyelitis, or trauma without any radiation. Ossification time and influencing factors were evaluated.

**Results:**

Both duration of surgery (346 ± 90 min. vs. 498 ± 124 min.; p < 0.001) and hospitalization (8.7 ± 2.8 days vs. 13.4 ± 5.3 days, p = 0.006) were shorter in the MRONJ group compared to the noMRONJ group. Ossification after mandibular reconstruction was significantly faster in the MRONJ study group [224 days, interquartile range (IQR) 175–287] compared to the control group (288 days, IQR 194–445; p < 0.001). Moreover, good initial contact between the segments resulted in faster ossification (p < 0.001) in the MRONJ group. Ossification rate between original and grafted bone or between grafted bone segments only did not differ in both the study and control groups (MRONJ, p = 0.705 vs. control, p = 0.292). The type of antiresorptive agent did not show any significance for ossification. The rate of wound healing disturbances did also not differ between the study and control groups (p = 0.69).

**Conclusion:**

Advanced MRONJ (stage 3) can be resected and reconstructed safely with free microvascular bone flaps. Antiresorptive agents enhance the ossification of the bone segments. Optimal initial contact of the bone segments accelerates bone healing. Surgery and hospitalization are markedly shortened in this vulnerable group of MRONJ patients compared to oncologic patients.

## Introduction

First used against hypercalcemia, bisphosphonates became an important adjuvant drug for patients suffering from bone metastases (1). Nowadays, the group of antiresorptive drug agents includes the group of bisphosphonates and denosumab (2).

Bisphosphonates interact with the osteoclasts, leading to inhibition and suppression of bone resorption. After incorporation orally or intravenously, the agent binds to hydroxyapatite and can be detected after more than one decade (3). Denosumab, however, is a monoclonal antibody, which prevents the binding of the receptor activator of nuclear factor-κB (RANK) and RANK ligand (RANKL). By docking RANKL on RANK, osteoclast precursor cells become stimulated and lead, among others, to the differentiation of the osteoclasts. This control mechanism plays a major role in bone remodeling, and by being specifically blocked, bone resorption is reduced (4).

With worldwide age-standardized incidence rates (ASIR) in 2020 of 47.8 per 100,000 for breast cancer (female) and 30.7 per 100,000 for prostate cancer (male), the two most common malignant tumors separated by gender enable a large group of patients to be treated with these medications (5, 6).

In addition to the treatment of bone metastases, antiresorptive therapy is well-established for multiple myeloma. With more than 150,000 cases a year worldwide in 2020, these patients set an additional part of the oncological prescriptions of antiresorptive drugs (5–7).

Moreover, the prevention of osteoporotic fractures has become a major socioeconomic factor in a worldwide aging population (8). With reduced doses and enlarged time intervals compared to the malignant indications, all antiresorptive agents are approved for osteoporotic treatment (9).

With the spread of bisphosphonates, a severe complication, the bisphosphonate-related osteonecrosis of the jaw (BRONJ), appeared and accumulated (10, 11). Later with anti-RANKL therapy, similar complications were observed and renamed to medication-related osteonecrosis of the jaw (MRONJ) (12). In general, patients with oncological indications have therefore a higher risk for MRONJ than those with osteoporosis (13).

Over the years, resection of the necrotic bone and stable, stressless soft tissue wound closure have turned out to be the key steps for curing MRONJ (14, 15).

In a very advanced stage of MRONJ (usually stage 3), segmental resection of the mandible may become necessary. Immediate reconstruction with free vascularized bone grafts has evolved as a feasible and reliable option (16, 17).

In addition to vascular flap complications or surgical site infections, non-union of the grafted bone segments presents a further important complication of free bone grafting. Proper osseous healing of the grafted bone is decisive for successful mandibular rehabilitation and the precondition for the removal of osteosynthesis material. Modulating bone metabolism antiresorptive agents are supposed to influence the ossification of reconstructed mandibles with free bone grafts.

Small case studies reported the feasibility and successful ossification of microvascular bone reconstructions in cases with stage 2 and 3 BRONJ (18, 19). However, no single study to date has presented more detailed data about the time and quality of bone ossification after free bone graft transfer.

Therefore, the aim of this retrospective study was to investigate the timeline and influencing factors for ossification after mandibular reconstruction with free vascularized bone grafts in MRONJ in comparison to a control group without antiresorptive agents or radiotherapy.

## Patients and methods

The study design was approved by the ethics committee of the University of Regensburg (ref. 23–3559-104) in accordance with the Declaration of Helsinki and its later amendments or comparable ethical standards.

### Patients

Between 2009 and 2022, 38 operations with free microvascular bony reconstructions of the mandible were identified in the Department of Oral and Maxillofacial Surgery at the University Hospital in Regensburg and were included in this study. Exclusion criteria were pre- or postoperative radiation therapy and no follow-up postoperative panoramic radiograph in the first 12 months. Four reconstructed patients dropped out because of missing follow-ups. Moreover, regular follow-ups with panoramic radiographs starting from 2 months until 2 years after reconstruction were available. Three different types of microvascular bony reconstruction were performed. The predominant type of reconstruction was the free fibula flap (n = 33), followed by microvascular iliac crest (n = 3) and scapular flaps (n = 2). No patient received two separate independent free flaps.

The following data were collected (**Tables 1A–C**): age, gender, diagnosis, antiresorptive agent, duration of dose delivery, preoperative computer-aided design/computer-aided manufacturing (CAD/CAM) planning, defect classification after Jewer and Boyd (20), type of bony reconstruction, amount and length of the bone segments, duration on intensive care unit (ICU) and duration of hospitalization, previous operations in the head and neck region, wound healing disorders, and revisions regarding the free graft.

**Table 1A:** Cohort characteristics for both groups.

	MRONJ group (MRONJ) (n = 13)	Control group (noMRONJ) (n = 25)
Male/female, n (%)	6 (46%)/7 (54%)	15 (60%)/10 (40%)
Age in years, median (IQR)	67 (62, 72)	53 (40, 62)
Radiation dose in Gy	none	none
CAD/CAM planning, n (%)	2 (15%)	4 (16%)
Neck dissection, n (%)	0 (0%)	11 (44%)
Previous operations in head/neck region	10 (77%)	12 (48%)
Disorder of postoperative wound healing	5 (39%)	8 (32%)
Revisions	6 (46%)	7 (28%)

MRONJ, medication-related osteonecrosis of the jaw; CAD/CAM, computer-aided design/computer-aided manufacturing; IQR, interquartile range.

**Table 1B:** Primary diagnosis for both groups and information about antiresorptive agents.

	MRONJ group (MRONJ) (n = 13)	Control group (noMRONJ) (n = 25)
Primary diagnosis		
Malignant tumor	0	12
MRONJ	13	0
Unspecific osteomyelitis	0	9
Gunshot wounds	0	2
Benign tumor	0	2
Type of antiresorptive agent		
Bisphosphonate	7	
Denosumab	3	
Combination	3	
Indication for antiresorptive agent		
Metastases by breast cancer	4	
Metastases by prostate cancer	3	
Metastases by kidney cancer	1	
Breast cancer without metastases	1	
Multiple myeloma	4	

MRONJ, medication-related osteonecrosis of the jaw.

**Table 1C:** Defect classification and types of microvascular flap utilized.

	MRONJ group (MRONJ) (n = 13)	Control group (noMRONJ) (n = 25)
Defect classification of Boyd and Jewer		
C	0	1
H	1	1
HC	0	1
HCL	0	1
HL	0	0
L	4	6
LC	1	9
LCL	7	6
Type of flaps		
Fibula	11	22
Iliac crest	0	3
Scapula	2	0

MRONJ, medication-related osteonecrosis of the jaw.

The cohort was divided into two groups regarding the primary reason for the reconstruction of the mandible: one study group with MRONJ and one control group (noMRONJ).

**Figure 1:**
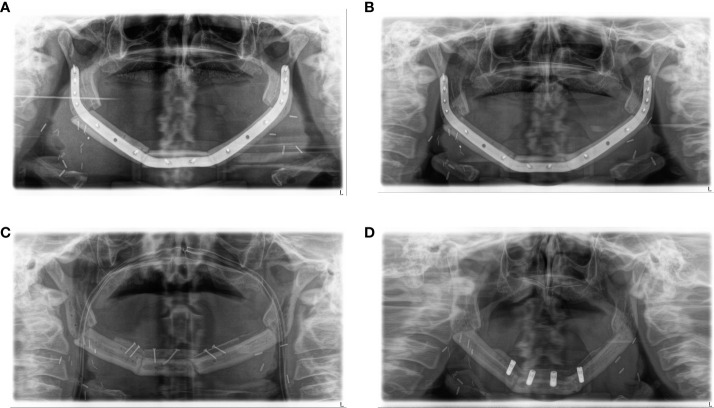
Example of ossification under antiresorptive agent: **(A)** patient with severe MRONJ of the mandible, resection, and reconstruction with a three-segmented fibula CAD/CAM-planned. **(B)** After 5 months, complete ossification in all four contact points. **(C)** Two months later, after removal of reconstruction plate and augmentation with avascular iliac crest. **(D)** Thirteen months after microvascular reconstruction and insertion of dental implants. MRONJ, medication-related osteonecrosis of the jaw; CAD/CAM, computer-aided design/computer-aided manufacturing.

**Figure 2:**
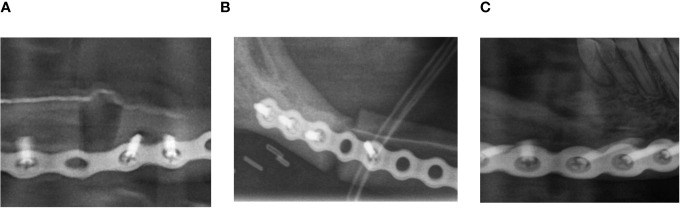
Example of evaluation of the initial contact point. **(A)** Example of no initial contact (radiographic gap >2 mm). **(B)** Example of moderate initial contact (radiographic gap ≤2 mm). **(C)** Example of good initial contact (no radiographic gap).

All available postoperative panoramic radiographs were examined (**Figure 1**). Location and quality of contact (no contact, moderate contact, and good contact) between the bone graft and original bone as well as between graft segments were documented and associated with healing time after surgery (**Figure 2**).

The segment number and segment length of the bone graft were evaluated, too.

Panoramic radiographs around the seventh postoperative day and the 6th, 11th, and 21st postoperative months were examined. After full ossification at each contact point, no further examinations were made.

Two raters (T.E. and M.G.) examined all radiographs independently and valued every point of contact with a three-part score. The raters faced a choice between no ossification (no sign of ossification vertically between the segments), partial ossification (less than 50% ossification between the segments), and complete ossification (more than 50% ossification between the segments). In the case of diverse evaluation between the raters, a review was performed, and a final statement was expressed.

### Data analysis

Continuous data are presented as mean ± SD or as median (first quartile, third quartile) depending on the underlying distribution and were compared between groups using a t-test for independent samples or the non-parametric Kruskal–Wallis test. Revisions and wound healing disturbance were analyzed by chi-square tests. Time to ossification was analyzed using Kaplan–Meier plots and log-rank tests as well as a multivariable Cox proportional hazards model, including all significant variables of the univariable analyses. Hazard ratios (HRs) and corresponding 95% confidence intervals (95% CI) are reported as effect estimates. A p-value <0.05 was considered statistically significant for all tests. All statistical analyses and plots were performed using IBM SPSS Statistics, version 29 (IBM Corp., Armonk, NY, USA).

## Results

The first panoramic radiograph was performed around the seventh postoperative day (7.4 ± 5.9) as a starting point and was used for evaluation of the primary contacts between the segments. Mean surveillance periods were at three different postoperative check-up dates. The first was around the sixth month (170 ± 64 days), the second around the 11th month (320 ± 92) days, and the last control X-ray around the 21st month (629 ± 233 days) after surgery. Overall, 114 points of contact between the segments were documented in panoramic radiographs. The duration of the operation was 498 ± 124 minutes significantly longer in the noMRONJ subgroup than the MRONJ subgroup, p < 0.001 (346 ± 90 minutes). The average postoperative stay in ICU showed no significant difference (noMRONJ subgroup 4.0 ± 3.4 days vs. MRONJ group 2.5 ± 1.6 days, p = 0.14). The control group had a significant (p = 0.006) longer overall hospitalization with 13.4 ± 5.3 days compared to the MRONJ group with 8.7 ± 2.8 days. The defect size showed no significant difference (p = 0.51) between the groups. The control group had a smaller average defect with 102 ± 37 mm in relation to the MRONJ group with 110 ± 36 mm. Revisions (p = 0.117) and wound healing disturbance (p = 0.69) showed no significant difference between the subgroups (noMRONJ vs. MRONJ).

### Overall ossification MRONJ compared to the control group

**Figure 3:**
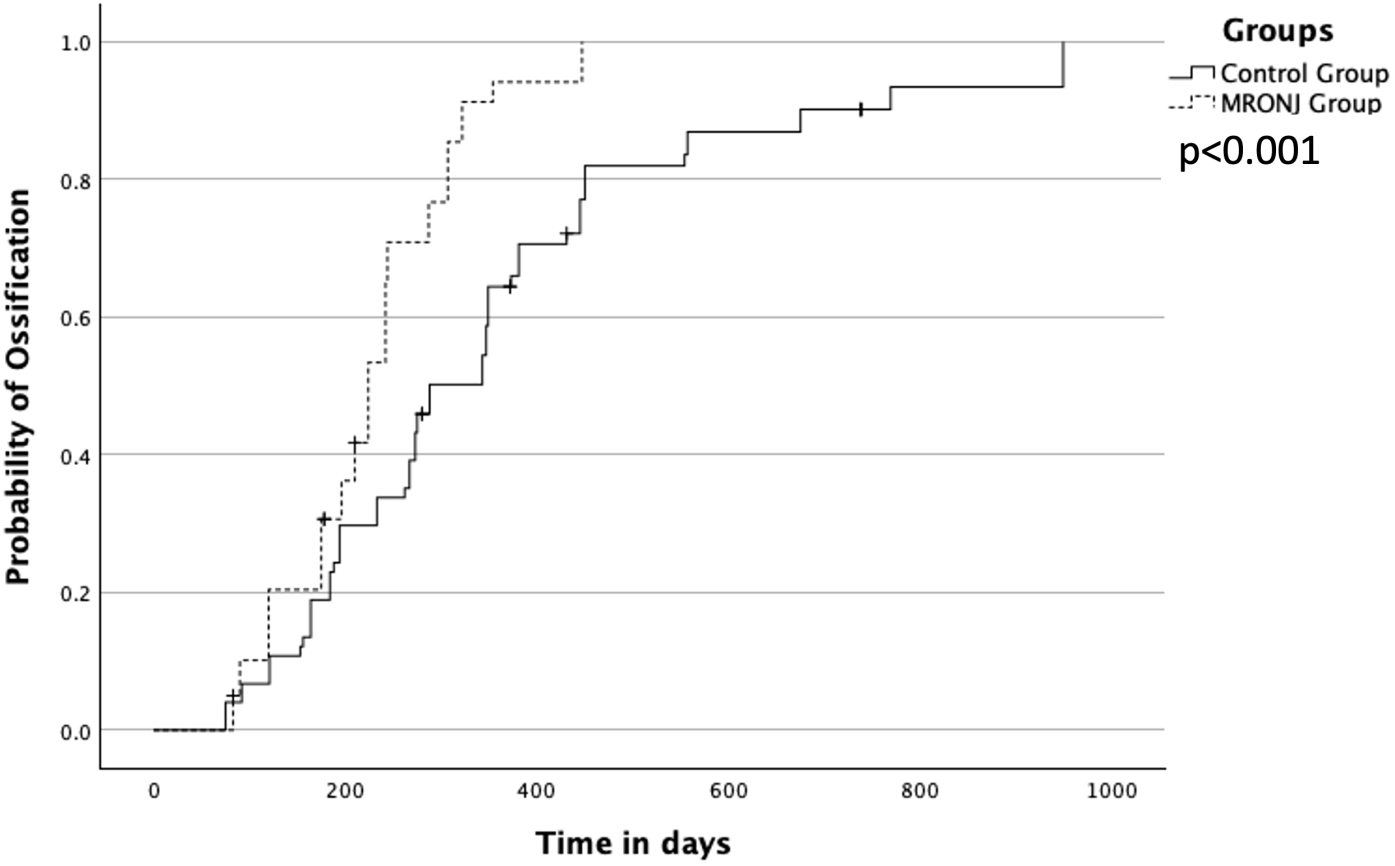
Overall ossification between MRONJ group and the control group (noMRONJ). MRONJ, medication-related osteonecrosis of the jaw.

The overall median for complete ossification at the point of contact was 273 days (interquartile range (IQR) 184–373). The fastest ossification was observed in patients with antiresorptive agents MRONJ in a median of 224 days (IQR 175–287). Patients without these agents showed 288 days (IQR 194–445). The difference between both groups was significant with p < 0.001 (**Figure 3**).

### Ossification concerning the bone quality

In the control group noMRONJ, the median ossification of contact points between two segments of the microvascular transplant showed was 273 (IQR 188–381) days, a slightly faster ossification than the ossification between one segment of the transplant and the original recipient bone of 343 (IQR 194–450) days.

**Figure 4:**
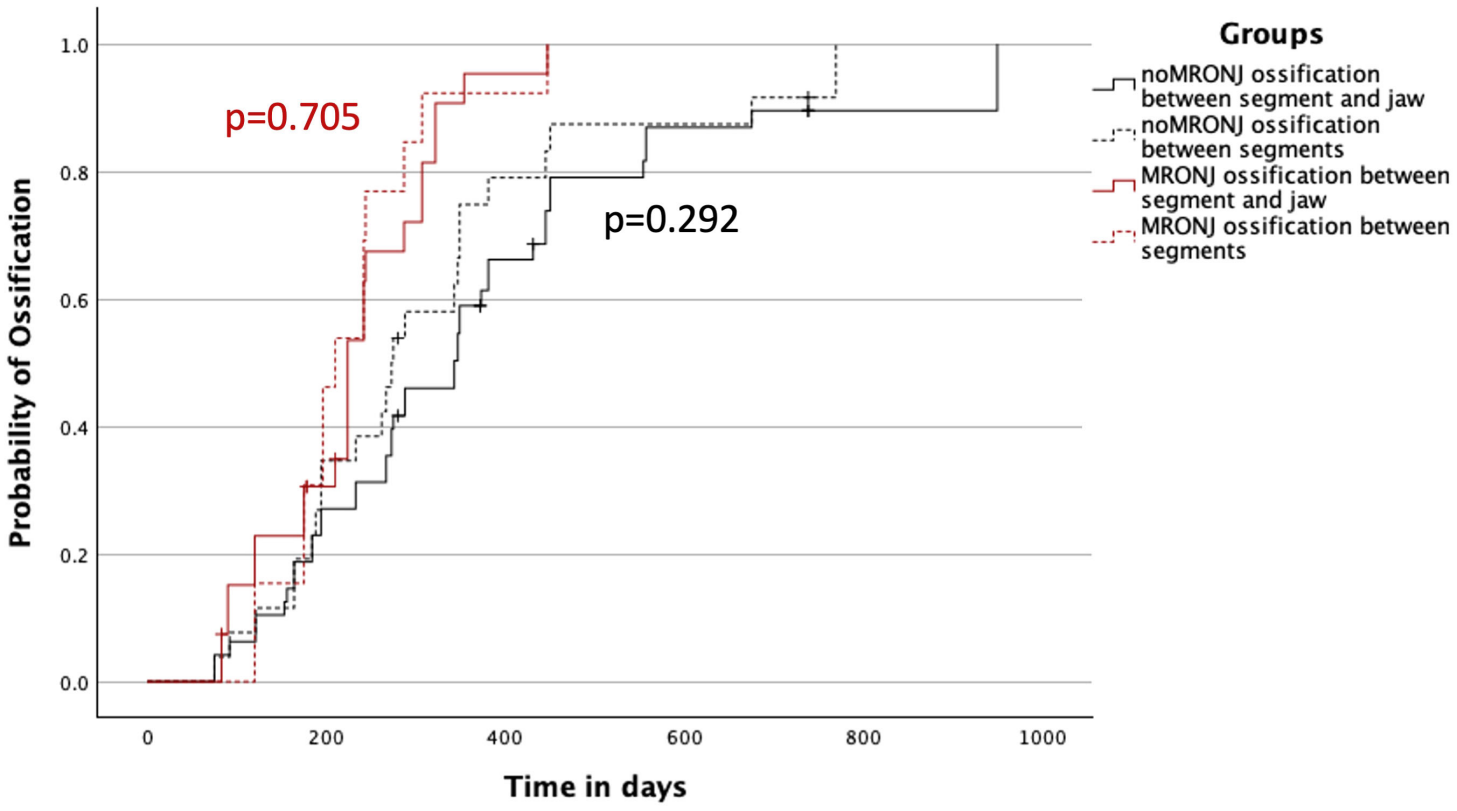
Ossification concerning the contact between two segments of the transplant and between one segment of the transplant and the original bone of the jaw divided into both MRONJ and noMRONJ groups. MRONJ, medication-related osteonecrosis of the jaw.

A similar distribution was observed in the MRONJ group with 210 (IQR 175–244) days for median ossification between transplanted segments and 224 (IQR 175–307) days for contact points between original bone and transplant. In both groups, the difference was not significant (noMRONJ, p = 0.292; MRONJ, p = 0.705) (**Figure 4**).

### Influence of initial contact between the bone segments on ossification and analysis of multiple variables regarding ossification

**Figure 5:**
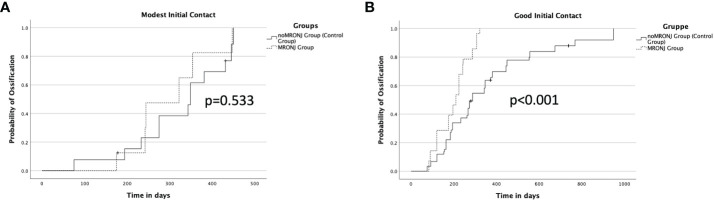
**(A)** Ossification concerning a modest initial contact between both groups. **(B)** Ossification concerning good initial contact between both groups.

The quality of the initial contact showed an impact on ossification between noMRONJ and MRONJ. While a moderate initial contact showed, p = 0.533, no faster ossification between noMRONJ (median 349 days, IQR 275–431) and MRONJ (median 322 days, IQR 242–354) (**Figure 5A**), a good initial contact could demonstrate significant, p < 0.001, faster ossification for the MRONJ group with a median of 210 days (IQR 120–242) compared to noMRONJ with 288 days (IQR 184–450) (**Figure 5B**).

**Figure 6:**
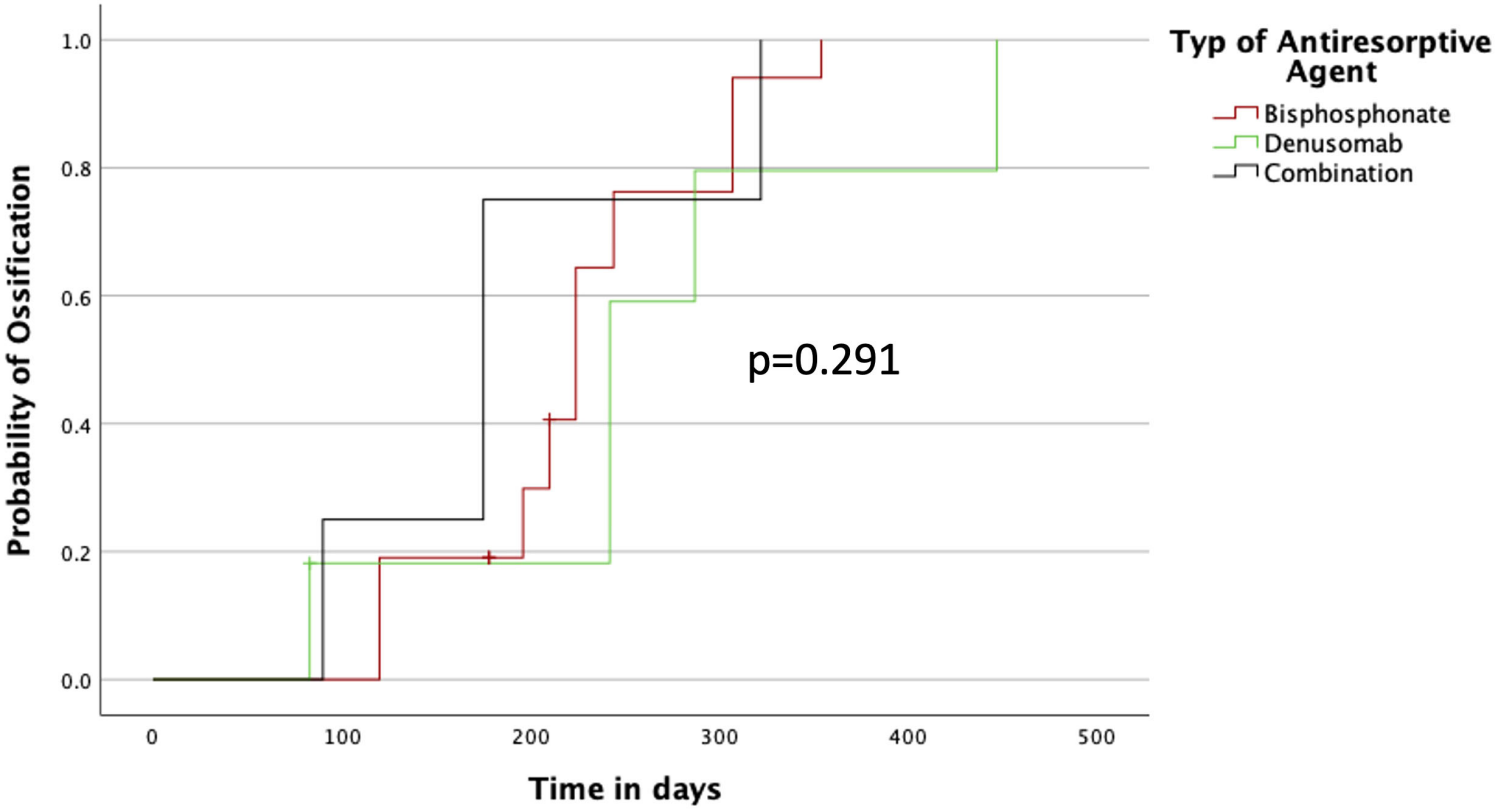
Ossification concerning the type of antiresorptive agent.

By analyzing the subgroup MRONJ, the effects of different antiresorptive agents were examined. The fastest ossification was observed by patients having combined therapy of bisphosphonate and denosumab within 175 days (IQR 90–175), followed by patients with only bisphosphonates within 224 days (IQR 196–244) and patients with only denosumab therapy within 242 days (IQR 242–287) without significance, p = 0.291 (**Figure 6**).

Moreover, all cofactors were examined in a multivariable Cox regression model on ossification. Only the main variable of antiresorptive agent showed a significant impact on ossification [HR (95% CI), 4.71 (2.36; 9.39), p < 0.001] (**Table 2**).

**Table 2:** Multivariable Cox regression model on ossification.

	HR	95% CI		p-Value
**No history of antiresorptive agents**	4.705	2.359	9.386	**<0.001**
**Contact between transplant segments**	1.365	0.890	2.095	0.154
Quality of initial contact				
No initial contact	Reference			
Moderate initial contact	1.412	0.439	4.545	0.563
Good initial contact	2.430	0.801	7.368	0.117
**Gender**	0.986	0.581	1.673	0.959
**Age**	0.989	0.975	1.004	0.144
**No tobacco abuse**	1.256	0.676	2.332	0.471
**No alcohol abuse**	0.932	0.470	1.849	0.840
**CAD/CAM planning**	0.687	0.357	1.323	0.262
**Prior operations in the head and neck region**	0.600	0.357	1.009	0.054
Revision				
No revision	Reference			
One revision needed	1.219	0.733	2.027	0.445
Multiple revisions needed	0.683	0.240	1.939	0.474

CAD/CAM, computer-aided design/computer-aided manufacturing. Values in bold indicate statistically significant results.

## Discussion

The influences of antiresorptive agents on the metabolism of the bone are well known, but the effects on the ossification of bony reconstructions of the mandible have not been investigated in detail so far. The aim of this study was to evaluate the bony healing after mandibular reconstruction with free microvascular bone grafts in patients with advanced MRONJ. During follow-up, panoramic radiographs revealed a reliable ossification in most contact points of the reconstructed mandible. Moreover, ossification was significantly faster compared to the control group (noMRONJ) consisting of free bone graft reconstructions due to tumor or osteomyelitis without a history of antiresorptive medication or radiation therapy.

The effects on indirect fracture healing have already been examined in animal models. Rats treated with alendronate showed faster radiographic healing. In biomechanical testing, the callus even showed higher stability than in the control group. Nevertheless, remodeling processes of the callus were delayed in comparison to those in animals of the control group (21).

Similar results could be achieved in a rabbit model for the examination of bone healing after mandibular fractures under zoledronate, leading to accelerated bone healing with higher stability compared to the control group (22). These results support the outcome of the current study with faster ossification for patients with antiresorptive agents in their history. In this context, human studies on patients with fractures and bisphosphonate intake showed no delay in bony healing under antiresorptive medication (23).

The safe reconstruction of the mandible via free microvascular bone graft in patients with MRONJ has been proven in the past (16, 24). Described fistulas or postoperative infections could be treated with antibiotics or minor surgical revisions in most cases; very rarely is removal of osteosynthesis necessary. Stable intra- and extraoral soft tissue closure remains a key prerequisite according to general MRONJ treatment recommendations (15).

Both groups of drug agents, bisphosphonates and denosumab, affect the bone remodeling of the whole skeleton (25). Therefore, this study showed no significant difference in bone healing between grafted segments and between grafted and mandibular segments. This differs from irradiated patients with significantly faster bone healing between the non-irradiated grafted segments (26). The slightly faster ossification of intersegmental graft contact points—as found in the current study—may be attributed to a better matching contact surface compared to an incongruent graft/mandibular angle junction.

For both subgroups (noMRONJ and MRONJ), the initial contact defines the progress of ossification, and a good initial bone contact is warranted for predictable bony union and stable mandibular reconstruction (27). The differences between both subgroups appear significantly only in cases with good initial contact and is therefore an important goal for the surgical procedure. Higher accuracy of CAD/CAM-planned microvascular reconstructions of the mandible may lead to better initial contact and therefore may additionally improve ossification (28). However, patient-specific manufactured reconstruction plates may even delay ossification due to higher rigidity (29). The pooled analysis could not find a statistically significant difference in non-union between CAD/CAM and conventional planned cases (30).

The variable pharmacokinetics of bisphosphonates and denosumab are well documented. While integrated into the bone, the half-life of bisphosphonates is up to 10–12 years; meanwhile, the half-life of denosumab with binding RANKL is only 24–26 days (25). The switch from bisphosphonates to denosumab is still under investigation. Due to the overlap of the pharmacokinetics, synergistic effects of both medications on the metabolism of the bone seem likely (31). These tendencies may be seen in the different types of antiresorptive agents.

**Figure 7:**
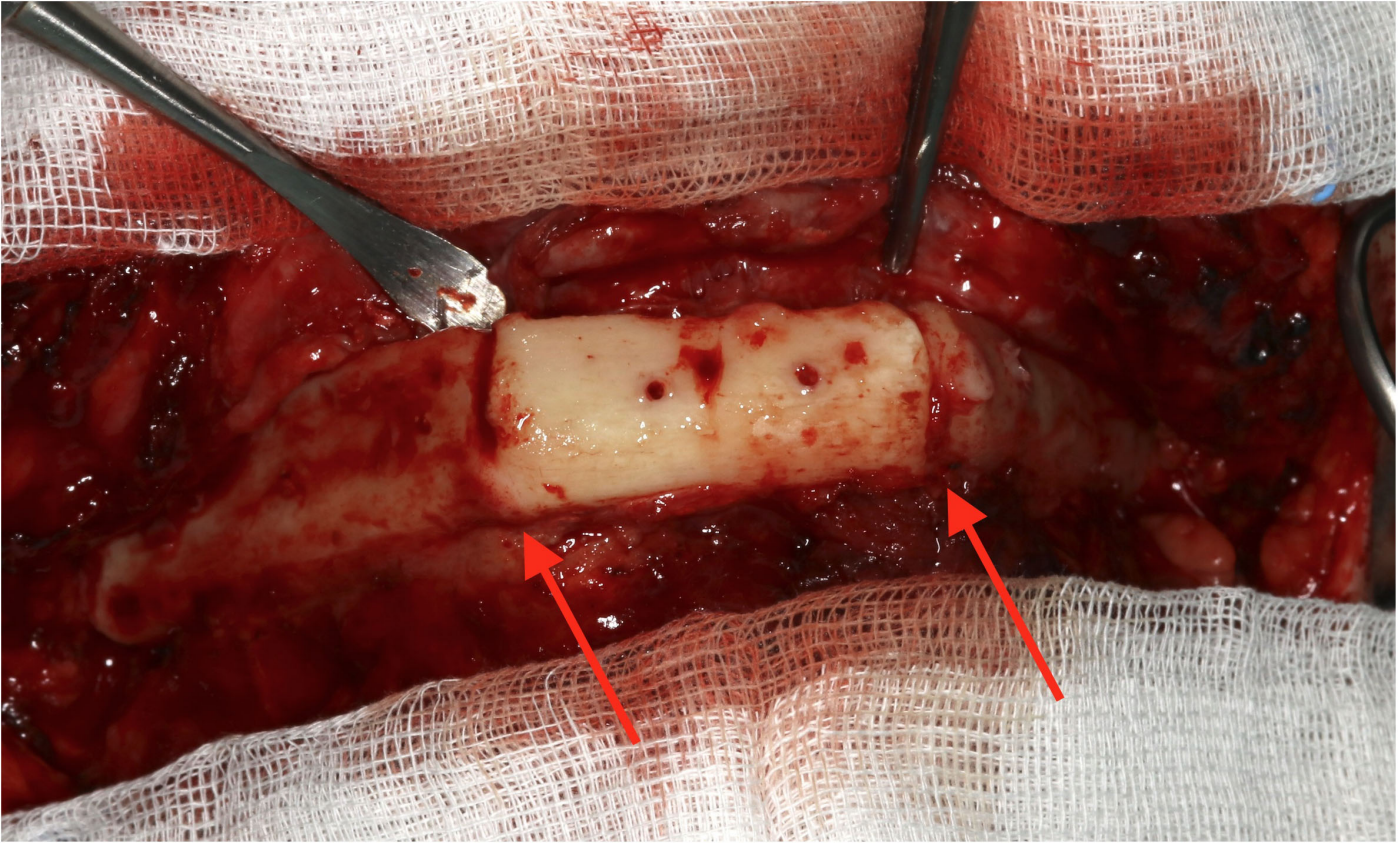
Intraoperative example of clinical complete ossification during removal of a reconstruction plate and before augmentation 7 months after microvascular reconstruction.

This study is based on a limited number of clinical cases, and imaging examinations were performed using panoramic X-rays instead of 3D radiographs like CT or cone-beam computer tomography (CBCT). Important associations may miss statistical significance due to missing power. With regard to imaging, post-surgical panoramic X-rays are performed in clinical routine after mandibular reconstructions, whereas 3D diagnostics are preserved for special indications such as mandibular reconstructions including temporomandibular joint (TMJ). Of course, 3D diagnostics are supposed to enable a more exact determination of ossification. Additionally, our grading of ossification relies on X-ray imaging. The gold standard would be a clinical validation. However, the removal of the reconstruction plate was performed rarely in our department (**Figure 7**).

At this point, it must be mentioned that although showing faster ossification, the MRONJ group came up with more revisions and wound healing disorders compared to the control group but without significant differences. Bisphosphonates not only affect bone metabolism but also have been shown to reduce cell viability, reduce proliferation, and increase apoptosis in oral keratinocytes and fibroblasts. Moreover, bisphosphonates have been demonstrated to reduce epithelial thickness and prevent epithelial formation in three-dimensional tissue-engineered models of the oral mucosa (32).

A further important aspect of this group of compromised patients is to keep reconstructive surgery short. This study clearly shows that fibula reconstruction is safely possible with successful bony healing within 5 to 6 hours of surgery. Surgery in MRONJ patients can be performed faster because typical oncological steps such as extended tumor resection and neck dissection are not necessary. In comparison with infected osteoradionecrosis (IORN), mandibular reconstruction in MRONJ patients is easier, as typical radiation-induced vessel fibrosis is absent, facilitating vascular anastomosis (33). Apart from technical ease, healing can be challenging in cases with IORN as well as MRONJ.

This study shows for the first time an enhanced ossification of microvascular mandibular reconstructions with free bone grafts in patients with advanced medication-related osteonecrosis of the jaw. Close initial segmental bone contact additionally accelerates ossification. A foregoing therapy with antiresorptive agents is no contraindication for major reconstructive surgery. Surgery in this group of compromised patients with jaw resection and free flap reconstruction can be safely performed with short recovery times.

## Data availability statement

The datasets presented in this article are not readily available because patients ID are part of the dataset. Requests to access the datasets should be directed to maximilian.gottsauner@ukr.de.

## Ethics statement

The studies involving humans were approved by ethics committee of the University of Regensburg (ref. 23-3559-104). The studies were conducted in accordance with the local legislation and institutional requirements. Written informed consent for participation was not required from the participants or the participants’ legal guardians/next of kin in accordance with the national legislation and institutional requirements.

## Author contributions

MG: Conceptualization, Data curation, Formal Analysis, Investigation, Methodology, Project administration, Resources, Visualization, Writing – original draft, Writing – review & editing. JM: Conceptualization, Investigation, Validation, Writing – review & editing. JE: Conceptualization, Validation, Writing – review & editing. SE: Conceptualization, Validation, Writing – review & editing. JS: Conceptualization, Validation, Writing – review & editing. MF: Conceptualization, Validation, Writing – review & editing. MM: Conceptualization, Validation, Writing – review & editing. TR: Validation, Writing – review & editing. TE: Conceptualization, Supervision, Validation, Writing – original draft, Writing – review & editing.
